# Bioactive chitosan packaging films with encapsulated beet leaf extract for preserving chicken fillets

**DOI:** 10.1016/j.fochx.2025.103335

**Published:** 2025-11-29

**Authors:** Solmaz Pourbarghi Soufiani, Shima Yousefi, Masoud Honarvar, Weria Weisany, Gholamhassan Asadi

**Affiliations:** a Department of Food Science, SR.C., Islamic Azad University, Tehran, Iran; b Department of Agriculture Science and Engineering, SR.C., Islamic Azad University, Tehran, Iran

**Keywords:** Active packaging, Chitosan composite films, Meat preservation, Nanoparticles, Plant extract

## Abstract

Extending the shelf life of perishable foods like chicken meat while minimizing synthetic preservatives and plastic waste is a key sustainability goal. This study developed an edible, film composed of chitosan (CH), cellulose nanocrystals (CNC), and beet leaf extract (BE) encapsulated in mesoporous silica nanoparticles (MSNPs). Beet leaves, a rich source of phenolics and natural pigments, were valorized into a nanocarrier system to enhance antioxidant protection and controlled release. Structural analyses (FT-IR, SEM, TEM) confirmed successful integration of all components. During 8-day refrigerated storage, the optimized film (2 % CH, 2 % MSNPs, 1 % CNC, 15 % BE) effectively limited oxidative and microbial spoilage, maintaining meat quality by moderating pH, reducing oxidation, and suppressing microbial growth below spoilage thresholds. Compared to control samples, films containing the full bioactive matrix demonstrated superior preservation effects. This multifunctional packaging system offers a scalable, eco-friendly solution for meat protection through synergistic integration of natural bioactives and nanotechnology.

## Highlights

• Development of biodegradable chitosan films reinforced with CNCs and MSNPs.
• Beet leaf extract was successfully encapsulated in SBA-15 mesoporous silica nanoparticles.
• Active films reduced lipid oxidation, microbial growth, and color deterioration in meat.
• The nanocomposite films extended chicken fillet shelf life during refrigerated storage.
• Sustainable packaging valorized agricultural waste and enhanced food preservation.

## 1 Introduction

Animal-derived food products, particularly poultry, are essential components of the human diet, offering high-quality proteins, essential amino acids, vitamins, and minerals (Cuchillo-Hilario et al., 2024). Among them, chicken meat is widely consumed globally due to its nutritional value, affordability, and low fat content (Erdaw & Beyene, 2022). However, chicken is also highly perishable because of its high moisture content, rich protein profile, and neutral pH, all of which provide ideal conditions for microbial proliferation and enzymatic activity (Sharma et al., 2022). These factors accelerate spoilage processes such as lipid oxidation and protein degradation, leading to undesirable changes in color, texture, odor, and flavor, and the potential formation of harmful compounds. Consequently, the development of effective preservation strategies is crucial to maintain the quality, safety, and shelf life of chicken meat during storage and distribution.

Conventional packaging materials, mainly derived from synthetic polymers such as polyethylene (PE), polypropylene (PP), and polyethylene terephthalate (PET), have been widely used for protecting perishable foods (Baneshi et al., 2024). Although these plastics offer excellent mechanical strength and barrier properties, they are non-biodegradable and contribute significantly to environmental pollution and plastic waste accumulation (Kelaniyagama et al., 2024). In response to increasing environmental and regulatory pressures, there has been a growing shift toward the development of sustainable, biodegradable, and active packaging systems that reduce environmental impact while enhancing food quality and safety (Ahmed et al., 2022).

Biodegradable edible films, formulated from natural polymers such as polysaccharides (chitosan, cellulose), proteins (gelatin, casein), and lipids, have emerged as viable alternatives to synthetic plastics (Gupta et al., 2022). These materials are biocompatible, renewable, and biodegradable, and can be engineered to carry bioactive agents such as antioxidants, antimicrobials, and nutrients(Ribeiro et al., 2021). Chitosan, in particular, has gained considerable attention due to its excellent film-forming ability, natural antimicrobial properties, and compatibility with a wide range of bioactives (Díaz-Montes & Castro-Muñoz, 2021). However, to enhance the performance of such films, functional reinforcement and controlled delivery systems are required.

Natural plant extracts and essential oils rich in phenolic compounds, flavonoids, and pigments are increasingly incorporated into edible films due to their antioxidant and antimicrobial properties (Deng, Wang, et al., 2024; Khubiev et al., 2023). Yet, their direct application presents several challenges: many are volatile, hydrophobic, photosensitive, or chemically unstable, which can limit their effectiveness in packaging systems. Therefore, encapsulation technologies have been employed to protect these bioactives and improve their stability, bioavailability, and controlled release.

This study advances existing strategies in active packaging by integrating three established approaches chitosan-based films, CNC reinforcement, and plant-extract encapsulation via MSNPs into a single multifunctional system, thereby achieving synergistic improvements not previously reported in similar films. While earlier works have incorporated plant extracts and essential oils rich in phenolics, flavonoids, and pigments into edible films for their antioxidant and antimicrobial properties (Deng, Wang, et al., 2024; Khubiev et al., 2023), these bioactives often suffer from volatility, hydrophobicity, and chemical instability, which reduce their efficacy during storage and processing. In contrast, the present study introduces a novel nanocarrier biopolymer hybrid design, where MSNPs encapsulate BE to protect sensitive compounds and enable controlled, sustained release of antioxidants and antimicrobials. Simultaneously, CNC reinforcement enhances the film's mechanical strength and barrier integrity, while chitosan contributes intrinsic antimicrobial activity and structural cohesion. This integrated approach overcomes the limitations of direct extract incorporation, resulting in a high-performance bioactive film with superior stability, functionality, and preservation capacity compared to conventional encapsulated plant-extract systems.

SBA-15 is a well-characterized mesoporous silica nanoparticle (MSNP) synthesized through a sol–gel process using the triblock copolymer Pluronic P123 as a structure-directing agent under acidic conditions. It possesses a highly ordered two-dimensional hexagonal mesoporous structure with uniform pore sizes typically ranging from 5 to 30 nm, high surface area (>600 m^2^/g), large pore volume, and thick silica walls, making it thermally and mechanically stable. These features, along with the abundance of silanol (Si–OH) groups on its surface, enable high loading capacity and strong interactions with encapsulated compounds (D. Zhao et al., 1998). Among various encapsulating carriers, MSNPs particularly SBA-15 are notable for their ordered pore structure, high surface area, thermal and mechanical stability, and biocompatibility (Ghaferi, Koohi Moftakhari Esfahani, Raza, Al Harthi, Ebrahimi Shahmabadi, & Alavi, 2021). These features allow them to effectively load and protect sensitive bioactives while enabling sustained release under food storage conditions (Akonjuen & Aryee, 2023). Although MSNPs have been extensively studied in biomedical and pharmaceutical contexts, their application in edible packaging materials remains limited.

In parallel, the food industry has shown growing interest in valorizing agricultural byproducts and food waste as sources of functional ingredients. One such underutilized resource is beet leaves (*Beta vulgaris* L.), which are typically discarded during sugar beet processing. These leaves are rich in phenolic acids, flavonoids, betalains, vitamins, and amino acids, making them promising candidates for natural antioxidants and preservatives (Thiruvengadam et al., 2024). Utilizing beet leaf extract (BE) not only supports waste valorization but also aligns with consumer demand for clean-label, plant-based packaging materials with health-promoting properties. Beet leaves are an abundant agro-industrial byproduct generated in large quantities during beet processing, yet they remain largely underutilized and often simply discarded as waste (Ebrahimi et al., 2024). Notably, these leaves are rich in bioactive phytochemicals (polyphenols and betalain pigments) and exhibit potent antioxidant and antimicrobial activities (de Carvalho et al., 2025), making their extracts promising as natural additives for incorporation into biodegradable food packaging films to enhance shelf life and inhibit spoilage (de Carvalho et al., 2025). Such valorization of beet leaf waste not only mitigates agricultural waste but also aligns with the growing consumer demand for “clean-label” packaging solutions that utilize plant-based, non-synthetic materials (Cristofoli et al., 2023).

The integration of BE-loaded SBA-15 nanoparticles into chitosan-based edible films, reinforced with cellulose nanocrystals (CNCs), presents a novel strategy for the development of multifunctional, sustainable food packaging. CNCs serve as structural reinforcements that enhance mechanical strength, barrier performance, and film integrity, while maintaining biodegradability and transparency (Antony Jose et al., 2025; Deng, Hu, et al., 2024; Stark, 2016). This synergistic combination of materials enables the creation of films with enhanced oxidative stability, antimicrobial activity, and extended shelf life for perishable foods.

In recent years, the development of active edible films using natural bioactives and biodegradable polymers has gained momentum as a sustainable strategy for extending the shelf life of perishable foods (Daeialiakbar et al., 2025). Chitosan, a biopolymer with inherent antimicrobial and film-forming properties, has been widely explored in food packaging applications (Kumar et al., 2020). However, its functional performance can be further enhanced through the incorporation of nanostructured materials and bioactive compounds. While plant extracts rich in phenolics have been previously used in chitosan films, their direct incorporation often suffers from instability and uncontrolled release, limiting their efficacy. To address these challenges, encapsulation strategies have emerged as effective tools for stabilizing sensitive bioactives and enabling controlled release. This study investigated the encapsulation of BE, a rich source of phenolic compounds, into SBA-15-type MSNPs and their incorporation into a chitosan-based film reinforced with CNCs. The use of SBA-15, characterized by its ordered mesoporous structure, high surface area, and tunable pore size, enabled the controlled integration and delivery of bioactives within the film matrix. The integration of BE-loaded SBA-15 into chitosan films, further strengthened with CNCs, creates a multifunctional matrix with improved physicochemical, antioxidant, and antimicrobial properties. To our knowledge, this is the first report that combines these three components BE, SBA-15, and CNCs within an edible biopolymer film for meat preservation. This innovative platform offers promising potential for the development of eco-friendly active packaging solutions targeting oxidative and microbial spoilage in poultry products.

The integration of BE-loaded SBA-15 into chitosan films, reinforced with CNCs, results in a multifunctional, biodegradable matrix with enhanced physicochemical, antioxidant, and antimicrobial properties. While similar edible films have been studied, the combined use of SBA-15 nanocarriers, CNCs, and a valorized beet leaf extract in a chitosan matrix represents a novel approach not previously reported for meat preservation. Importantly, all film components chitosan, CNCs, and mesoporous silica are increasingly accessible through scalable and food-compatible processes, and have shown regulatory promise in food-contact applications. Recent advances in extrusion, solvent casting, and roll-to-roll coating technologies further support the industrial feasibility of producing such composite films. Thus, beyond laboratory innovation, this formulation offers a realistic platform for eco-friendly active packaging solutions with commercial potential in refrigerated meat supply chains.

This study aimed to develop and characterize multifunctional bioactive edible films composed of chitosan, CNCs, and SBA-15 MSNPs encapsulating BE, and to evaluate their effectiveness in extending the shelf life of chicken fillets under refrigerated storage. The research was guided by two central questions: (1) Does encapsulation of BE within SBA-15 nanoparticles improve its functional stability and efficacy compared to direct incorporation? and (2) Do nanocomposite films combining encapsulated BE with CNC reinforcement exhibit synergistic improvements in antioxidant, antimicrobial, and barrier performance relevant to food preservation? Based on these questions, we hypothesized that: (i) SBA-15 encapsulation would enable controlled release and protection of BE's phenolic compounds, leading to sustained bioactivity during storage; and (ii) integrating CNCs would enhance the film's structural and barrier properties, further improving meat quality preservation outcomes. The films were systematically assessed for physicochemical performance, antioxidant and antimicrobial activities, and their ability to inhibit lipid and protein oxidation, microbial proliferation, and sensory degradation over time. The novelty of this study lies in its first-time integration of valorized beet leaf extract, SBA-15 nanocarriers, and CNCs into a chitosan-based biopolymer film, establishing a multifunctional delivery platform for natural, active packaging. This approach supports sustainable packaging development and offers new insight into structure-function relationships in bioactive film design, with relevance to commercial poultry preservation and clean-label food packaging innovation.

## 2 Material and methods

### 2.1 Synthesis of Mesoporous Silica Nanoparticles SBA-15

SBA-15 were synthesized using a hydrothermal method based on the self-assembly of tetraethyl orthosilicate (TEOS) and Pluronic P123 (EO20PO70EO20) under acidic conditions. Initially, 30 mL of 37 % hydrochloric acid was mixed with 138 mL of deionized water in a reaction vessel. To this acidic solution, 6.0 g of Pluronic P123 was added and stirred until fully dissolved, allowing the formation of micellar templates. Subsequently, 13.7 g of TEOS was rapidly added to 50 mL of deionized water and immediately introduced into the Pluronic P123 solution under vigorous stirring. The resulting mixture was continuously stirred at 40 °C for an additional 30 min to promote precursor hydrolysis and condensation. The homogeneous solution was then transferred into a Teflon-lined autoclave and subjected to hydrothermal treatment at 100 °C for 48 h. Upon cooling to room temperature, the resulting solid product was recovered by vacuum filtration using Whatman No. 1 filter paper. To remove residual reactants and acidic by-products, the collected solid was thoroughly washed with deionized water until the pH of the filtrate reached the neutral range (pH 7–8). The purified white precipitate was subsequently dried overnight at 60 °C and calcined at 550 °C for 5 h in air to remove the organic surfactant template, resulting in the formation of well-ordered SBA-15 mesoporous silica nanoparticles (Thahir et al., 2019).

### 2.2 Beet Leaf Extract

Three sugar beet (*Beta vulgaris*) varieties Pirola, Puma, and BTS505 were selected for extract preparation. The collected leaves were thoroughly washed with distilled water to remove dirt and surface impurities, then air-dried at room temperature (∼25 °C) for 48 h. After drying, the leaves were finely ground into a uniform powder using a laboratory grinder. The powdered material was stored in sealed containers at 4 °C, protected from light until extraction. For the extraction process, 55 g of the dried leaf powder was soaked in 70 % ethanol (*v*/v) for 72 h at ambient temperature. The mixture was stirred occasionally to enhance the release of phenolic and bioactive compounds. After extraction, the mixture was filtered through Whatman No. 1 filter paper using a Buchner funnel to separate the plant residue from the liquid phase. The resulting filtrate was concentrated and evaporated to dryness under reduced pressure using a rotary evaporator (EYELA N1000, Tokyo, Japan) at a temperature not exceeding 45 °C. The dried beet leaf extract was collected and stored in airtight vials at 4 °C until further use in encapsulation and characterization experiments.

### 2.3 Analysis of Beet Leaf Extract

The chemical composition of the beet leaf extract was analyzed using high-performance liquid chromatography coupled with mass spectrometry (HPLC-MS) equipped with an electrospray ionization (ESI) source. The analysis was performed using a WATERS ALLIANCE 2695 HPLC system coupled with a Micromass Quattro micro API triple quadrupole mass spectrometer. Chromatographic separation was achieved on a C18 reverse-phase column (4.6 × 150 mm, 5 μm) maintained at 35 °C. The binary mobile phase consisted of solvent A (water with 0.1 % formic acid) and solvent B (acetonitrile with 0.1 % formic acid), delivered at a flow rate of 0.3 mL/min. The injection volume was 5 μL. Mass spectrometric detection was performed in both positive and negative ion modes under the following conditions: source temperature 120 °C, desolvation temperature 350 °C, capillary voltage 4.0 kV, cone voltage 25 V, RF lens voltage 0.2 V, and nitrogen as the desolvation gas at 200 L/h. Data acquisition and processing were conducted using MassLynx software. Compounds were identified by comparing retention times and mass fragmentation patterns with those of authentic standards, and where standards were not available, tentative identification was based on MS/MS spectra in comparison with literature and public databases. Quantification was performed using calibration curves generated from structurally similar compounds (Medic et al., 2022).

### 2.4 Microencapsulation of Extract in SBA-15 Nanoparticles

For the microencapsulation process, 100 μL of Triton X-100 was first added to microtubes containing 20 mg of SBA-15 nanoparticles to enhance dispersibility. Subsequently, 2000 μL of the previously prepared beet leaf extract was introduced into each tube. The mixture was stirred continuously using a magnetic stirrer at 550 rpm for 4 h to facilitate adsorption of the extract into the mesoporous structure of the silica. After incubation, the suspension was centrifuged at 10,000 rpm for 10 min to separate the encapsulated particles from the solution. The supernatant was carefully removed, and the solid pellet was left to dry overnight at room temperature to obtain the extract-loaded SBA-15 nanocomposites (Weisany, Samadi, Tahir, Amini, & Hossaini, 2022).

### 2.5 Encapsulation Efficiency (EE)

To evaluate the EE of phenolic compounds within the SBA-15 nanoparticles, 100 mg of the dried extract-loaded capsules were suspended in 1 mL of an ethanol–methanol mixture and vortexed briefly to ensure uniform dispersion. The suspension was then centrifuged at 10,000 rpm for 10 min, and the free (non-encapsulated) phenolic content in the supernatant was quantified using the Folin–Ciocalteu colorimetric method. Absorbance readings were measured spectrophotometrically, and the results were expressed as milligrams of gallic acid equivalents (GAE) per gram of dry weight (Luca, 2012). The encapsulation efficiency was calculated using the following equation:

%EE = [(Total phenolic content − Free phenolic content) / Total phenolic content] × 100

### 2.6 Morphological and Structural Analysis of SBA-15 Nanoparticles

The surface morphology and internal structure of the synthesized SBA-15 were characterized using scanning electron microscopy (SEM) operated at 30 kV and transmission electron microscopy (TEM) at 100 kV. These imaging techniques provided detailed insights into the particle size, shape, and pore organization, confirming the successful synthesis of well-ordered, uniformly distributed mesoporous structures. To evaluate the successful encapsulation of beet leaf extract into the SBA-15 framework, Fourier-transform infrared spectroscopy (FT-IR) was performed in the spectral range of 400–4000 cm^−1^. Comparative analysis of FT-IR spectra from empty SBA-15, pure extract, and extract-loaded SBA-15 revealed characteristic shifts and variations in peak intensities corresponding to specific functional groups, indicating effective incorporation of the extract into the silica matrix (Sattari et al., 2020).

### 2.7 Preparation of Films

Composite films were prepared using the solvent casting method. To formulate the chitosan-based film matrix, 2 g of chitosan powder was dissolved in 98 mL of 2 % (*v*/v) aqueous acetic acid under continuous magnetic stirring at 500 rpm for 90 min at room temperature (∼25 °C). After full dissolution, 0.5 g of glycerol was added as a plasticizer, and the mixture was stirred for an additional 30 min at the same speed to ensure uniform dispersion. The resulting film-forming solution was then poured into polystyrene casting plates (260 × 260 mm) and dried at 35 °C for 12 h in a controlled environment to produce uniform films (Hu et al., 2022).

CNCs were prepared separately via acid hydrolysis from microcrystalline cellulose. Specifically, 10 g of microcrystalline cellulose was suspended in 90 mL of deionized water, then hydrolyzed using 64 % sulfuric acid at 44 °C for 2 h. The suspension was subsequently centrifuged multiple times at 12,000 rpm for 15 min, with intermittent washing using distilled water until the pH of the suspension reached approximately 5. The final product was filtered through Whatman No. 1 filter paper to obtain a purified CNC suspension (Celebi & Kurt, 2015). Film samples were prepared by incorporating varying proportions of chitosan, MSNPs, CNC, and BE extract, as outlined in Table 1. The formulations were designed to systematically assess the influence of each component on the mechanical, barrier, antioxidant, and antimicrobial properties of the films. Following comprehensive evaluation, the formulation exhibiting the best overall performance characterized by high structural integrity, low permeability, and superior bioactive retention was identified as the optimized film. A control film containing no active components was also prepared to serve as a baseline for comparative analysis.

**Table 1:** Composition (%) of optimized film formulations.

Sample Code	% PVA	% Chitosan	% MSNPs	% Cellulose	% Beet Extract
Control	0	0	0	0	0
1	17	0	0	0.5	5
1.5	17	0	0	1	10
2	17	2	0	1	10
3	21	2	2	0.5	15
4	24	2	2	1	15

#### 2.7.1 Water vapor transmission rate (WVTR)

The water vapor transmission rate (WVTR) of the films was measured according to the gravimetric cup method based on ASTM E96–95, with slight modifications. Circular film samples (diameter 50 mm) were carefully cut and sealed over the open mouth of aluminum test cups containing 10 mL of distilled water to maintain 100 % relative humidity (RH) inside the cup. The assembled cups were weighed and placed in a controlled chamber maintained at 25 ± 1 °C and 50 ± 2 % RH. The cups were weighed every 12 h for 72 h, and the rate of weight loss (ΔW/Δt) was determined from the linear portion of the weight loss versus time curve. The WVTR (g·m^−2^·s^−1^) was calculated using the following equation:

$$\text{WVTR}=\frac{\Delta W}{A\cdot \Delta t}$$

where *ΔW* is the weight change (g) over time *Δt* (s), and *A* is the film's exposed area (m^2^).

#### 2.7.2 Oxygen transmission rate (OTR)

The oxygen transmission rate (OTR) was determined following ASTM D3985–05 using a gas permeability tester (Mocon Ox-Tran 2/21, USA) equipped with a coulometric oxygen sensor. Film samples were conditioned at 25 ± 1 °C and 50 ± 2 % RH for 24 h prior to analysis. During testing, the film separated two chambers: one side was continuously purged with high-purity oxygen (100 %), and the other side with nitrogen as the carrier gas. The steady-state oxygen flux passing through the film was recorded after equilibrium was reached, typically within 6–8 h. The OTR (cm^3^·m^−2^·day^−1^·Pa^−1^) was automatically calculated by the instrument and normalized to film thickness and test area.

### 2.8 In Vitro Release Study of Beet Leaf Extract from SBA-15 Nanoparticles

The release behavior of BE encapsulated within MSNPs was evaluated under simulated physiological conditions to assess its sustained antioxidant delivery potential. Briefly, 50 mg of BE-loaded SBA-15 was suspended in 50 mL of phosphate-buffered saline (PBS, pH 7.4) and incubated in a shaking water bath at 37 ± 0.5 °C with constant agitation at 100 rpm. At predetermined intervals (0, 2, 4, 8, 12, 24, 36, 48, 72, and 96 h), 2 mL aliquots of the release medium were withdrawn and immediately replaced with fresh PBS to maintain sink conditions.

The concentration of BE released into the medium was quantified by UV–Vis spectrophotometry at 425 nm, corresponding to the characteristic absorbance maximum (λ_max) of beet-derived betalain pigments. A calibration curve was established using known concentrations of BE in PBS. Cumulative release (%) was calculated by dividing the amount of BE released at each time point by the total BE content encapsulated in the SBA-15 particles. All measurements were performed in triplicate, and results are reported as mean ± standard deviation.

### 2.9 Preparation of Chicken Fillets

Fresh skinless chicken breast fillets (Pectoralis major) from broiler chickens (Ross 308, 5–6 weeks old) were sourced from a certified local poultry farm in Tehran, Iran, which operates under standard commercial rearing practices. Slaughter was conducted on the same day as the experiment under hygienic conditions at an approved facility. The fillets were transported to the laboratory within 2 h under chilled conditions using insulated containers with ice packs to maintain a temperature below 4 °C. Upon arrival, the fillets were trimmed, cleaned, and cut into uniform 100 g portions to ensure consistency across all experimental groups. Each sample was then individually packaged using the prepared active or control films. The packaged fillets were stored at 4 ± 1 °C and analyzed for physicochemical, oxidative, and microbiological changes on Days 1, 4, and 8 of refrigerated storage.

### 2.10 pH

The pH of the chicken fillet samples was measured to assess spoilage progression during storage. For each sample, 10 g of chicken meat was homogenized with 90 mL of distilled water using a high-speed homogenizer at 13,000 rpm for 10 s to obtain a uniform mixture. The pH of the resulting homogenate was immediately measured using a calibrated digital pH meter (Vargas et al., 2011).

### 2.11 Hydrogen Peroxide Content

Hydrogen peroxide levels, an indicator of oxidative spoilage, were measured spectrophotometrically following the modified method described by (Erel, 2005). In brief, 1 g of homogenized chicken fillet was mixed with 10 mL of cold phosphate-buffered saline (PBS, pH 7.4) and centrifuged at 10,000 rpm for 10 min at 4 °C. The supernatant was collected for analysis. To quantify hydrogen peroxide, 100 μL of the supernatant was reacted with 900 μL of a colorimetric reagent consisting of xylenol orange (100 μM), ferrous ammonium sulfate (250 μM), sorbitol (25 mM), and sulfuric acid (0.25 M). The mixture was incubated at room temperature for 30 min in the dark, allowing hydrogen peroxide to oxidize Fe^2+^ to Fe^3+^, which then forms a complex with xylenol orange, producing a purple color. The absorbance of the reaction mixture was measured at 560 nm using a UV–Vis spectrophotometer. A standard curve was prepared using known concentrations of hydrogen peroxide (0–2 μmol/g), and the results were expressed as μmol of hydrogen peroxide per gram of sample (μmol/g).

### 2.12 Thiobarbituric Acid (TBA)

The TBA assay was conducted to evaluate secondary lipid oxidation by quantifying malondialdehyde (MDA), a major byproduct of fatty acid peroxidation. For each sample, 1 g of chicken meat was homogenized with 4 mL of 10 % TCA and 5 mL of 4 % perchloric acid at 8000 rpm to precipitate proteins and extract oxidized lipids. The homogenate was filtered, and 5 mL of 20 mM TBA solution was added to 5 mL of the filtrate. The reaction mixture was incubated in a water bath at 95 °C for 40 min to facilitate the formation of MDA–TBA complexes. After cooling to room temperature, the absorbance of the solution was measured at 532 nm using a spectrophotometer. TBA values were calculated and expressed as mg malondialdehyde (MDA) per kg of chicken meat using a standard curve prepared from 1,1,3,3-tetramethoxypropane (TEP) (Ouattara et al., 2002).

### 2.13 Total Volatile Basic Nitrogen (TVB-N)

The TVB-N content, an indicator of protein degradation and microbial spoilage, was determined using a steam distillation method. For each analysis, 10 g of minced chicken sample was mixed with 2 g of magnesium oxide (MgO) and 300 mL of distilled water in a Kjeldahl flask. A few boiling chips and an anti-foaming agent (n-octanol) were added to prevent bumping during distillation. The distillation was carried out until approximately 125 mL of distillate was collected in a receiving flask containing 25 mL of 3 % boric acid and a few drops of methyl red indicator. The distillate was then titrated with 0.1 N sulfuric acid (H₂SO₄) until the endpoint was reached, indicated by a clear color change from green back to red (Botta et al., 1984). The TVB-N value was calculated using the formula:

TVB-N (mg/100 g) = 14 × volume of sulfuric acid used (mL)

### 2.14 Fat Acidity Measurement

Fat acidity, an indicator of lipid hydrolysis and free fatty acid release, was determined by titration. A 5 g portion of the chicken sample was first extracted with hexane to isolate the lipid fraction. The hexane extract was then filtered to remove any solids, and the clear fat solution was transferred to a 50 mL volumetric flask. The extracted fat was titrated with 0.1 M sodium hydroxide (NaOH) using phenolphthalein as an indicator until a persistent pink endpoint was observed. The fat acidity was calculated and expressed as grams of oleic acid per 100 g of sample. All measurements were performed in triplicate to ensure accuracy and reproducibility (Arabpoor et al., 2021).

### 2.15 Protein Oxidation

Protein oxidation was assessed by quantifying carbonyl groups, which are formed as a result of oxidative modification of amino acid side chains. The method involved derivatization of carbonyls with 2,4-dinitrophenylhydrazine (DNPH), followed by spectrophotometric analysis at 370 nm. Samples were first precipitated using 10 % trichloroacetic acid (TCA) and centrifuged to collect the protein pellet. The pellet was then resuspended in 2 M hydrochloric acid (HCl) and reacted with DNPH for carbonyl derivatization. After incubation, the absorbance of the resulting hydrazone derivatives was measured at 370 nm using a UV–Vis spectrophotometer. The extent of protein oxidation was expressed as nanomoles of carbonyl per milligram of protein (nmol/mg protein), based on a standard calibration curve (Soglia et al., 2022).

### 2.16 **Microbio**log**ical Analysis**

Microbiological quality was assessed by determining the total viable count (TVC) and psychrotrophic bacterial count (PTC). For each sample, 10 g of chicken was aseptically transferred into a sterile stomacher bag and mixed with 90 mL of sterile buffered peptone water. The mixture was homogenized using a stomacher blender to ensure even microbial distribution. Serial dilutions were prepared, and 0.1 mL aliquots from appropriate dilutions were surface-plated on Tryptic Soy Agar (TSA). The plates were incubated at 35 °C for 48 h to determine the TVC, and at 4 °C for 10 days to assess the PTC. Colonies were counted and results were expressed as log CFU/g of sample (Arashisar et al., 2004).

### 2.17 Statistical analysis

Statistical analysis was conducted to determine the significance of differences in physicochemical, microbiological, and sensory parameters across different film formulations and storage periods (Days 1, 4, and 8). All data were analyzed using SAS software (version 9.2) following a two-way analysis of variance (ANOVA) to evaluate the main and interactive effects of formulation type and storage time. The experiment was arranged in a completely randomized design (CRD) with three independent replicates per treatment (*n* = 3). Mean values are presented as mean ± standard error (SE), and differences among means were compared using Duncan's multiple range test at a significance level of *p* < 0.05. This statistical approach ensured a reliable assessment of data variability, reproducibility, and treatment effects.

## 3 Result

### 3.1 Chemical compounds of beet leaf extracts

HPLC-MS analysis of beet leaf extracts revealed a rich diversity of bioactive chemical compounds, including phenolics, flavonoids, coumarins, and anthocyanins. Notable compounds identified across different extract formulations include 2,3-dihydroxy-1-guaiacylpropanone, pyrogallol, catechol, esculin, 4-ethylguaiacol, and coumarin each known for their antioxidant properties (Table 2). Flavonoid derivatives such as naringenin 7-O-glucoside, chrysoeriol 7-O-(6″-malonyl-apiosyl-glucoside), apigenin 7-O-(6″-malonyl-apiosyl-glucoside), and 7-hydroxymatairesinol were also prevalent, indicating strong potential for anti-inflammatory and antimicrobial activity. Additionally, anthocyanins like pelargonidin and peonidin glycosides, as well as cholesterol-derived ferulates, contributed to the antioxidant and color-enhancing attributes of the extracts. The presence of secoisolariciresinol-sesquilignan, theaflavin, and prodelphinidin trimers further underscores the extract's functional versatility, making beet leaf an excellent source of natural compounds for active food packaging and health-related applications.

**Table 2:** The chemical compounds found in the beet leaf extracts, along with their respective retention times as determined by liquid chromatography-mass spectrometry (LC-MS).

Compound (BTS1)	%	RT	Compound (Pirola)	RT	%	Compound (Poma)	RT	%
2,3-Dihydroxy-1-guaiacylpropanone	2.700	2.72	4-Ethylguaiacol	2.75	4.311	Coumarin	2.63	4.585
4-Hydroxycoumarin	8.594	2.83	Coumarin	2.63	2.515	Catechol	2.62	3.012
4-Ethylguaiacol	3.828	2.78	p-Anisaldehyde	2.85	2.612	7,4’-Dihydroxyflavone	2.82	2.183
Coumarin	2.827	2.67	Pyrogallol	2.64	3.103	Naringenin 7-O-glucoside	2.6	0.859
Pyrogallol	6.315	2.67	Pyrogallol	2.62	6.195	7-Hydroxymatairesinol	2.59	16.920
Naringenin 7-O-glucoside	0.903	2.66	Esculin	2.59	6.034	Esculin	2.6	7.662
5–5’-Dehydrodiferulic acid	2.970	2.68	2,3-Dihydroxy-1-guaiacylpropanone	2.68	4.168	3-Hydroxyphloretin 2’-O-glucoside	3.86	0.709
7-Hydroxymatairesinol	11.055	2.65	Catechol	2.77	1.835	5–5’-Dehydrodiferulic acid	2.63	4.928
Esculin	3.788	2.65	Ferulaldehyde	2.77	3.860	Ligstroside-aglycone	3.87	0.550
Chrysoeriol 7-O-(6″-malonyl-apiosyl-glucoside)	1.469	8.1	4-Hydroxycoumarin	2.79	8.424	Gallic acid ethyl ester	3.89	1.743
Luteolin 7-O-diglucuronide	1.638	8.08	5–5’-Dehydrodiferulic acid	2.61	3.239	Pyrogallol	3.14	7.062
Kaempferol 3-O-(6″-acetyl-galactoside) 7-O-rhamnoside	3.345	8.1	7-Hydroxymatairesinol	2.6	20.568	6-Prenylnaringenin	2.6	7.569
Pelargonidin 3,5-O-diglucoside	1.243	7.56	3-Caffeoylquinic acid	3.88	0.507	Spinacetin 3-O-glucosyl-(1- > 6)-glucoside	5.02	1.074
Peonidin 3-O-(6″-p-coumaroyl-glucoside)	1.345	8.39	Ligstroside-aglycone	3.84	0.487	Pelargonidin 3,5-O-diglucoside	7.03	2.420
24-Methylenecholestanol ferulate	2.905	7.43	Valoneic acid dilactone	3.2	0.500	Peonidin 3-O-(6″-p-coumaroyl-glucoside)	5.07	3.393
Theaflavin	6.543	7.47	Peonidin 3-O-(6″-p-coumaroyl-glucoside)	8.01	1.069	24-Methylcholesterol ferulate	8.82	1.181
Pelargonidin 3-O-arabinoside	2.338	9.42	24-Methylcholesterol ferulate	13.05	1.869	Pelargonidin 3-O-arabinoside	9.28	4.166
5,6-Dihydroxy-7,8,3′,4′-tetramethoxyflavone	0.768	9.46	1,2-Diferuloylgentiobiose	9.17	2.027	(+)-Gallocatechin	9.32	7.442
(+)-Gallocatechin	4.127	9.45	Pelargonidin 3-O-arabinoside	9.3	4.231	(+)-Catechin	8.61	1.511
Diosmin	6.471	8.4	(+)-Gallocatechin	9.33	3.046	Diosmin	7.98	4.602
Apigenin 7-O-(6″-malonyl-apiosyl-glucoside)	13.671	8.41	Apigenin 7-O-(6″-malonyl-apiosyl-glucoside)	8.09	15.074	Apigenin 7-O-(6″-malonyl-apiosyl-glucoside)	8.04	13.992
24-Methylcholesterol ferulate	1.914	13.11	p-Coumaroyl tartaric acid	13.13	0.866	Quercetin 3-O-(6″-malonyl-glucoside)	14.6	1.821
Delphinidin 3-O-feruloyl-glucoside	0.247	13.11	Cyanidin 3-O-(6″-malonyl-3″-glucosyl-glucoside)	22.21	2.112	Bisdemethoxycurcumin	9.31	0.616
Prodelphinidin trimer C-GC-C	0.374	13.21	Vitisin A	16.54	1.349			
24-Methylcholesterol ferulate	1.873	13.11						
Gallic acid 3-O-gallate	5.560	16.52						
Secoisolariciresinol-sesquilignan	1.034	16.61						
Cyanidin 3-O-(6″-malonyl-3″-glucosyl-glucoside)	0.156	22.32						

However, plant-based antioxidants are inherently unstable when exposed to light, heat, oxygen, or acidic/basic conditions (Mohammadi et al., 2025). To overcome this limitation, the extract was encapsulated in SBA-15 type MSNPs. These MSNPs offer a high surface area, tunable pore sizes, and an ordered porous structure, which together serve as an ideal vehicle for loading, protecting, and releasing bioactive molecules in a controlled manner (Xu et al., 2023). The interaction between silanol groups on the MSNPs and hydroxyl or carboxyl groups in phenolic compounds occurs through hydrogen bonding, allowing stable encapsulation (Sterman & Marsden, 1966; Weitkamp et al., 2021). Upon incorporation into the film matrix, the MSNPs facilitated the slow and sustained release of the extract's active compounds, thereby extending antioxidant activity throughout the storage period and preventing premature degradation.

### 3.2 Characterization of Mesoporous Silica Nanoparticles (MSNPs)

#### 3.2.1 Transmission Electron Microscopy (TEM) Analysis

**Fig. 1:**
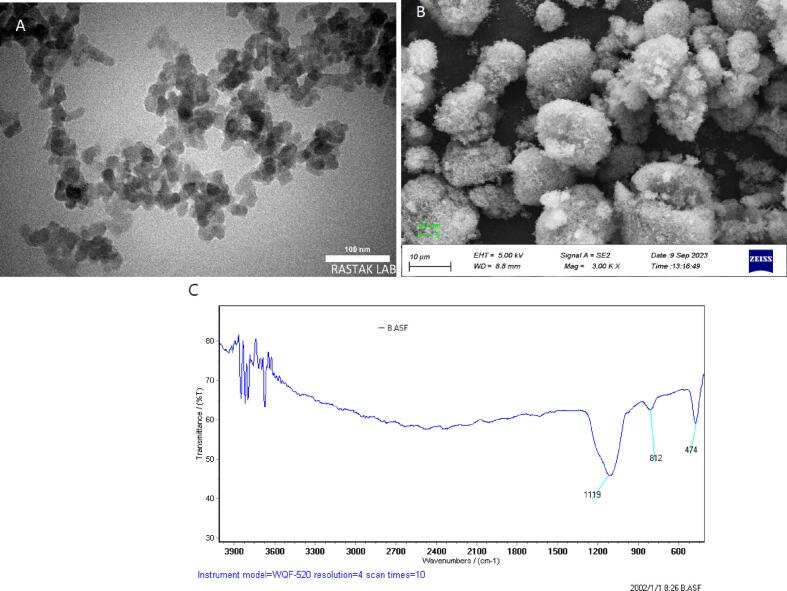
Characterization of mesoporous silica nanoparticles (MSNPs): (A) Transmission Electron Microscopy (TEM) image highlighting the surface morphology and particle distribution of the mesoporous silica structure. (B) Scanning Electron Microscopy (SEM) image highlighting the surface morphology and particle distribution of MSNPs. (C) Fourier Transform Infra-Red (FTIR) spectrum showing functional group analysis and confirmation of the MSNPs. (For interpretation of the references to color in this figure legend, the reader is referred to the web version of this article.)

TEM imaging (Fig. 1A) provided high-resolution visualization of the internal and surface morphology of MSNPs. The particles displayed a uniform, spherical shape with a clearly defined porous network, consistent with mesoporous structures. The well-ordered and radial pore arrangement observed within individual particles indicates the successful formation of a mesostructured silica framework. The contrast differences in the TEM images further support the presence of internal porosity, as the electron-transparent regions correspond to the mesopores. The overall size distribution appeared narrow, and no signs of agglomeration were visible, demonstrating good monodispersity and stable dispersion in the sample preparation.

#### 3.2.2 Scanning Electron Microscopy (SEM) Analysis

Complementary SEM analysis (Fig. 1B) revealed additional morphological details at the particle surface level. MSNPs were predominantly spherical with smooth surfaces and relatively consistent sizes, supporting the uniformity of synthesis. The particles were well-separated, indicating minimal aggregation and favorable colloidal stability. Although SEM provides less resolution of internal pore structure compared to TEM, the surface texture and particle arrangement seen in the SEM micrographs further corroborate the mesoporous nature of the synthesized MSNPs.

#### 3.2.3 **Fourier Transform Infrared (FTIR) Spectroscopy**

The chemical structure and functional groups of the MSNPs were examined by FTIR spectroscopy (Fig. 1C). The spectrum displayed several characteristic absorption peaks that confirm the successful synthesis of silica-based nanoparticles. A broad band centered around 3400 cm^−1^ corresponds to O—H stretching vibrations, indicative of surface hydroxyl groups or adsorbed water. The strong absorption band at approximately 1080 cm^−1^ is attributed to the asymmetric stretching of Si–O–Si bonds, while the peak at ∼800 cm^−1^ corresponds to symmetric Si–O–Si stretching. A smaller band near 460 cm^−1^ represents Si—O bending vibrations. The absence of additional peaks associated with organic contaminants suggests the purity of the MSNPs and confirms that no residual synthesis reagents remain.

Collectively, these findings confirm that the synthesized MSNPs possess a uniform, spherical morphology with a well-developed mesoporous structure and characteristic silica-based functional groups. The integration of TEM, SEM, and FTIR analyses provides robust validation of the successful fabrication and structural integrity of the MSNPs, making them suitable for further functionalization or application in nanomedicine and material science.

Chitosan, a cationic polysaccharide with film-forming ability, further enhanced the functionality of the films (Y. Zhao et al., 2023). Chitosan's positive charge allows it to interact with negatively charged microbial cell membranes, increasing membrane permeability and leading to leakage of intracellular components and eventual cell death (Tamara et al., 2018). In addition to this antimicrobial mechanism, chitosan's molecular structure enables it to form dense hydrogen-bonded networks that act as an effective barrier to oxygen and moisture both key accelerators of spoilage in high-protein, high-moisture foods like chicken (Manzoor et al., 2022; Singh et al., 2023). These interactions were confirmed by FTIR analysis, which showed notable shifts and increased intensities in peaks corresponding to O—H and N—H stretching, indicating strong molecular interactions among film components.

CNCs served as reinforcing agents that significantly improved the mechanical and barrier properties of the films (Aouay et al., 2023; Leppänen et al., 2022). Due to their crystalline nature, high aspect ratio, and abundance of surface hydroxyl groups, CNCs formed a hydrogen-bonded network within the polymer matrix, resulting in a tighter and more rigid structure (Antony Jose et al., 2025). This “tortuous path” mechanism reduced the diffusion rate of oxygen and water vapor through the film, contributing to the inhibition of oxidative reactions and microbial growth (Criado et al., 2020). SEM analysis revealed that films containing CNCs and chitosan had smoother and more compact surfaces, indicating improved structural integrity and reduced porosity compared to CNC-free films.

### 3.3 Water Vapor and Oxygen Transmission Rates

The barrier properties of the developed films were evaluated by measuring their water vapor transmission rate (WVTR) and oxygen transmission rate (OTR) (Table 3). Both parameters decreased progressively with the incorporation of chitosan, CNCs, MSNPs, and BE, indicating a denser and less permeable film structure. The control sample (without active components) exhibited the highest WVTR (1.23 × 10^−11^ g m^−1^ s^−1^ Pa^−1^) and OTR (7.02 × 10^−13^ g m^−1^ s^−1^ Pa^−1^), reflecting a weak barrier against moisture and gas diffusion.

**Table 3:** Water vapor transmission rate (WVTR) and oxygen transmission rate (OTR) of chitosan-based edible films containing different concentrations of mesoporous silica nanoparticles (MSNPs), cellulose nanocrystals (CNCs), and beet extract (BE).

PVA (%)	Chitosan (%)	MSNPs (%)	CNC (%)	Beet Extract (%)	Water vapor transmission rate (g m^−1^ s^−1^ Pa^−1^)	Oxygen transmission rate (g m^−1^ s^−1^ Pa^−1^)
0	0	0	0	0	1.23 ± 0.03 a	7.02 ± 0.09 a
17	0	0	0.5	5	1.18 ± 0.02 b	6.61 ± 0.08 b
17	0	0	1	10	1.14 ± 0.02 c	6.32 ± 0.07 c
17	2	0	1	10	1.11 ± 0.01 d	5.83 ± 0.06 d
21	2	2	0.5	15	1.09 ± 0.02 e	5.32 ± 0.05 e
24	2	2	1	15	1.07 ± 0.02 f	4.83 ± 0.04 f

Incorporation of CNCs alone (Films 1 and 1.5) significantly reduced (*p* < 0.05, *n* = 3) both the WVTR and OTR, likely due to the formation of a dense, hydrogen-bonded crystalline network. This structure effectively limited polymer chain mobility and introduced a tortuous diffusion pathway that hindered moisture and gas permeability. Further addition of chitosan (Film 2) enhanced these effects, lowering WVTR and OTR by approximately 10–15 % compared with CNC-only films, likely due to the polycationic nature of chitosan and its strong interfacial bonding with CNCs. The inclusion of MSNPs and higher BE concentrations (Films 3 and 4) resulted in the lowest permeability values (1.07 × 10^−11^ g m^−1^ s^−1^ Pa^−1^ for WVTR and 4.83 × 10^−13^ g m^−1^ s^−1^ Pa^−1^ for OTR), confirming a compact polymeric network with synergistic barrier reinforcement.

### 3.4 In Vitro Release Profile of BE Extract from SBA-15 Nanoparticles

**Fig. 2:**
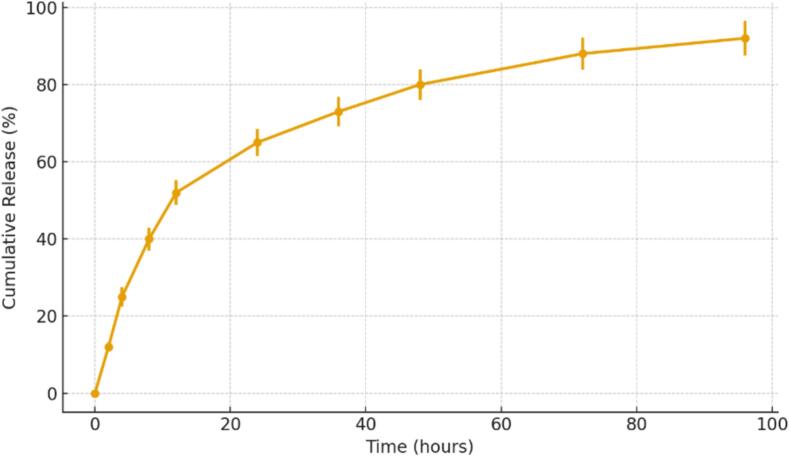
Simulated cumulative release profile of beet leaf extract (BE) from SBA-15 mesoporous silica nanoparticles over 96 h at ambient conditions. The data represent mean values with standard deviation (*n* = 3), illustrating a sustained and controlled release pattern characteristic of SBA-15's porous structure. Error bars indicate variability across replicates.

The release behavior of BE from MSNPs exhibited a sustained and gradual profile over 96 h (Fig. 2). An initial burst release of approximately 25 % was observed within the first 4 h, likely due to surface-adsorbed extract molecules loosely bound to the external pore walls. This was followed by a slower, controlled release phase, reaching 65 % release at 24 h and approximately 92 % by the end of the 96-h incubation period. The progressive release pattern indicates effective encapsulation and retention of BE within the nanoporous structure of SBA-15, consistent with its high surface area and well-ordered pore architecture. The standard deviations across triplicate samples remained low (±4.5 % at maximum), demonstrating good reproducibility of the encapsulation and release process. These results support the potential of SBA-15 as a nanocarrier for controlled delivery of plant-derived antioxidants in food packaging applications.

### 3.5 pH

**Fig. 3:**
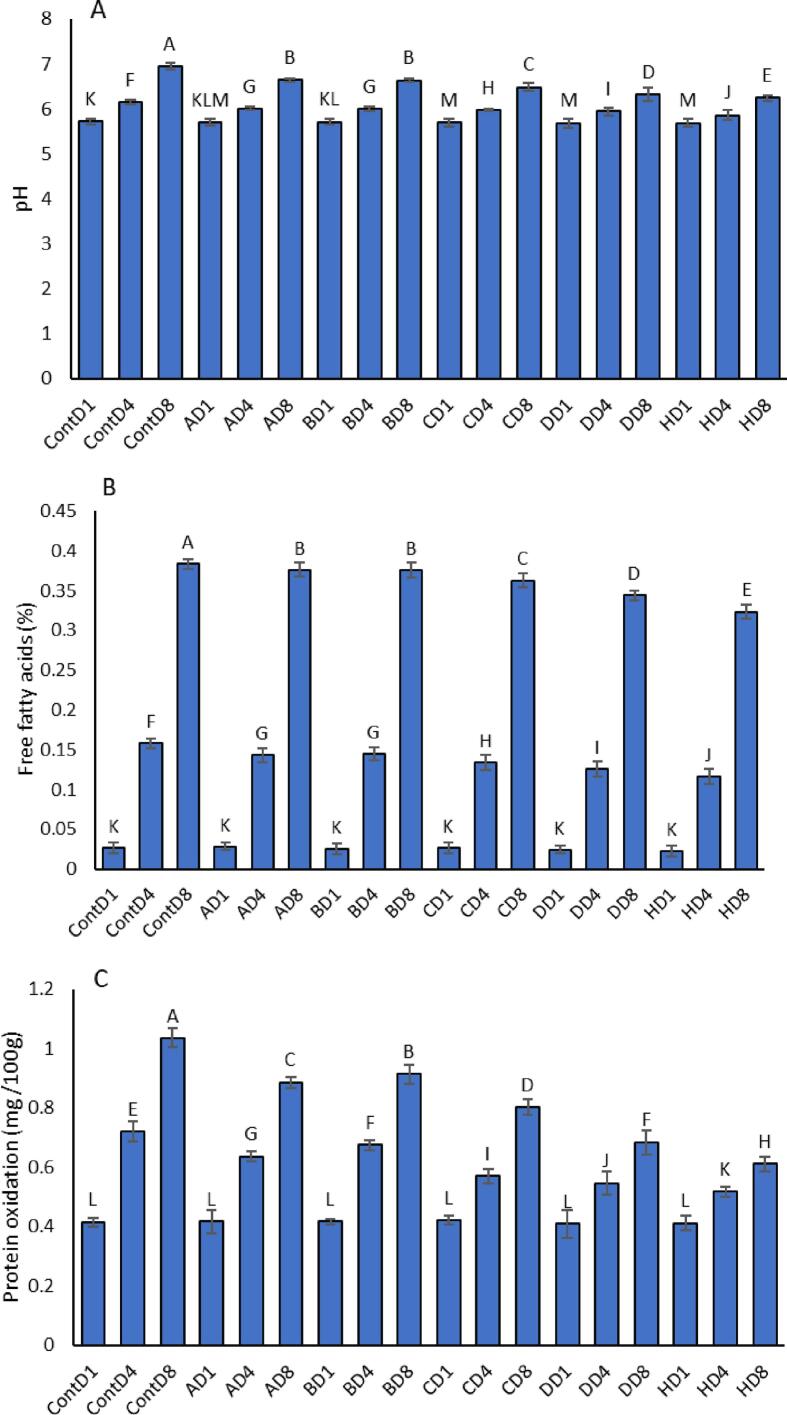
Effect of film composition on the pH (A), free fatty acids (B), and protein oxidation (C) of chicken fillet during refrigerated storage (Days 1, 4, and 8) to evaluate their potential for shelf-life extension. The formulations included: Control (0 % of all components), A (17 % PVA, 0 % chitosan, 0 % mesoporous silica, 0.5 % CNC, 5 % extract), B (17 % PVA, 0 % chitosan, 0 % mesoporous silica, 1 % CNC, 10 % extract), C (17 % PVA, 2 % chitosan, 0 % mesoporous silica, 1 % CNC, 10 % extract), D (17 % PVA, 2 % chitosan, 2 % mesoporous silica, 0.5 % CNC, 15 % extract), and H (17 % PVA, 2 % chitosan, 2 % mesoporous silica, 1 % CNC, 15 % extract). Data represent the mean ± standard error (SE) of three independent replicates (n = 3). Statistical significance was assessed using one-way ANOVA followed by Duncan's multiple range test at *p* < 0.05. Different lowercase letters above the bars (or data points) indicate significant differences among treatments, confirming the reliability and repeatability of the results.

As shown in Fig. 3A, the pH values of all chicken fillet samples gradually increased during the 8-day storage period, which is consistent with the production of alkaline compounds (e.g., amines and ammonia) due to microbial spoilage and protein degradation. The Control sample showed the most pronounced increase, starting from 5.72 on Day 1 and rising to 6.95 by Day 8, reflecting significant spoilage activity.

In comparison, active film formulations effectively moderated the pH rise, with clear differences depending on the film composition. Formulation H (17 % PVA, 2 % chitosan, 2 % mesoporous silica, 1 % CNC, and 15 % extract) demonstrated the greatest pH stability, increasing from 5.69 (Day 1) to 6.24 (Day 8), a much smaller shift compared to the Control. Similarly, formulations D and C, which also included chitosan and extract, showed controlled pH increases from 5.69 to 6.32 and 5.70 to 6.49, respectively. Films A and B, which lacked chitosan and mesoporous silica but contained lower amounts of extract (5–10 %) and CNC, showed intermediate effects. The pH of formulation A increased from 5.71 to 6.64, while formulation B rose from 5.71 to 6.63 over the 8-day period.

### 3.6 Free Fatty Acids (FFA)

As shown in Fig. 3B, the concentration of free fatty acids (FFA), a key indicator of lipid hydrolysis and spoilage, increased significantly in all treatments over the 8-day storage period. The Control group exhibited the highest FFA accumulation, rising from 0.027 % on Day 1 to 0.384 % on Day 8, confirming the extent of lipid degradation in the absence of active packaging. Among the active film treatments, formulation H demonstrated the most effective inhibition of FFA formation. FFA levels in this group increased modestly from 0.023 % (Day 1) to 0.323 % (Day 8) approximately 16 % lower than the Control at the end of the storage period. This suggests strong lipid-protective effects likely due to the combined antioxidant and barrier properties of chitosan, mesoporous silica, CNC, and extract.

Formulations D and C also reduced FFA levels by Day 8 to 0.344 % and 0.363 %, respectively, which were statistically lower than the Control group (0.384 %) based on two-way ANOVA followed by Duncan's multiple range test (*n* = 3, *p* < 0.05). Notably, these formulations also included chitosan and extract, further supporting the role of these components in minimizing lipid degradation. In contrast, films A and B, which lacked chitosan and mesoporous silica, exhibited higher FFA levels of 0.376 % at Day 8, nearly identical to the Control, indicating limited protective effects. These results highlight that the inclusion of chitosan and mesoporous silica, particularly in combination with natural extracts as in formulation H, is critical in retarding lipid hydrolysis and extending the lipid quality of chicken fillets during storage.

The integration of these components resulted in films that significantly delayed spoilage processes. Formulation H, which included 15 % BE, 2 % chitosan, 2 % MSNPs, and 1 % CNC, consistently outperformed other formulations in all preservation metrics. This film demonstrated the lowest levels of lipid oxidation (measured by TBARS and FFA), protein oxidation (carbonyl content), and hydrogen peroxide accumulation. These effects can be attributed to the combination of phenolic radical scavenging, oxygen restriction by CNCs, and the sustained release mechanism of MSNPs (Criado et al., 2017; Litescu et al., 2014). Similarly, microbial spoilage indicators such as total volatile nitrogen (TVN) and total bacterial count (TBC) were lowest in formulation H. This was likely due to the synergistic action of chitosan's membrane-disrupting effect, the antimicrobial properties of phenolics, and the protection and delivery function of MSNPs (Doan et al., 2025).

### 3.7 Protein Oxidation

Protein oxidation, measured via carbonyl content, increased in all chicken fillet samples over the 8-day storage period (Fig. 3C). This rise reflects oxidative stress and protein deterioration commonly associated with spoilage. The Control group exhibited the highest levels of oxidation, with carbonyl values rising sharply from 0.4151 (Day 1) to 1.0350 (Day 8) a 2.5-fold increase.

Formulations A and B, which lacked chitosan and mesoporous silica, showed only slightly improved protection, with Day 8 carbonyl contents reaching 0.886 and 0.913, respectively. Formulation C, which included 2 % chitosan and 10 % extract, showed a moderate improvement, limiting oxidation to 0.803 by Day 8. Significantly better results were observed with formulations D and H, both containing chitosan, mesoporous silica, CNC, and higher extract concentrations. Formulation D maintained carbonyl content at 0.685 by Day 8, while formulation H showed the most effective protection, limiting oxidation to just 0.611 roughly 41 % lower than the Control.

These results confirm that active films with enhanced antioxidant capacity especially those incorporating chitosan and high extract concentrations are effective in reducing oxidative damage to muscle proteins during refrigerated storage. Among them, formulation H demonstrated the most substantial inhibitory effect on protein oxidation.

The observed reduction in protein oxidation can be attributed to several mechanisms. First, chitosan possesses inherent antioxidant activity due to its polycationic nature, which enables it to chelate pro-oxidant metal ions and neutralize free radicals (Thambiliyagodage et al., 2023). Second, the beet leaf extract rich in phenolic compounds such as flavonoids and anthocyanins acts as a primary antioxidant by donating hydrogen atoms and stabilizing radical species. Third, encapsulation of the extract within the SBA-15 MSNPs protects these sensitive compounds from premature degradation and allows sustained release throughout storage. Additionally, CNC contributes to film integrity and acts as a barrier to oxygen diffusion (Wang et al., 2020). Together, these components create a multifunctional antioxidant matrix that effectively interrupts oxidative chain reactions and preserves protein structure during refrigerated storage.

### 3.8 Thiobarbituric Acid Reactive Substances (TBARS)

**Fig. 4:**
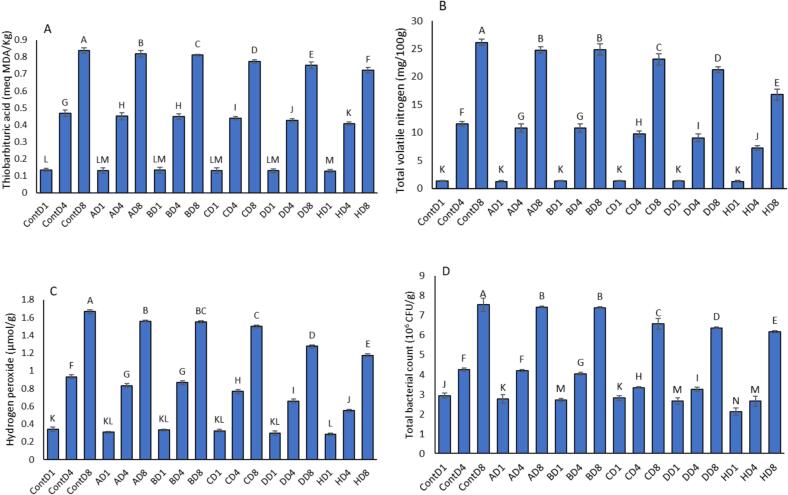
Effect of film composition on thiobarbituric acid reactive substances (TBARS, A), total volatile nitrogen (TVN, B), hydrogen peroxide content (C), and total bacterial count (D) of chicken fillet during refrigerated storage (Days 1, 4, and 8) to evaluate their potential for shelf-life extension. The formulations included: Control (0 % of all components), A (17 % PVA, 0 % chitosan, 0 % mesoporous silica, 0.5 % CNC, 5 % extract), B (17 % PVA, 0 % chitosan, 0 % mesoporous silica, 1 % CNC, 10 % extract), C (17 % PVA, 2 % chitosan, 0 % mesoporous silica, 1 % CNC, 10 % extract), D (17 % PVA, 2 % chitosan, 2 % mesoporous silica, 0.5 % CNC, 15 % extract), and H (17 % PVA, 2 % chitosan, 2 % mesoporous silica, 1 % CNC, 15 % extract). Data are presented as mean ± standard error (SE) of three independent replicates (n = 3). Statistical significance was determined using one-way ANOVA followed by Duncan's multiple range test at p < 0.05. Different lowercase letters above the bars (or data points) indicate statistically significant differences among treatments, demonstrating the reliability and reproducibility of the data.

TBARS values, which indicate the extent of secondary lipid oxidation, increased progressively across all treatments during the 8-day storage period (Fig. 4A). The Control group exhibited the highest TBARS accumulation, rising from 0.134 on Day 1 to 0.837 on Day 8, signifying a significant degree of lipid peroxidation in the absence of protective films. Formulations A and B showed marginal improvements, with Day 8 TBARS levels reaching 0.819 and 0.811, respectively very close to the Control. These two films lacked chitosan and mesoporous silica, which likely contributed to their limited antioxidative performance. More notable reductions were observed in formulations C and D, which included 2 % chitosan. Their Day 8 TBARS levels were 0.775 and 0.751, respectively. These results suggest that chitosan contributed to improved oxidative stability, although the difference remained moderate.

Formulation H was the most effective in suppressing TBARS formation, limiting values to 0.722 by Day 8. This formulation contained all active components chitosan, mesoporous silica, CNC, and 15 % extract providing a synergistic antioxidant barrier that significantly delayed lipid oxidation. Compared to the Control, formulation H reduced TBARS formation by approximately 14 % at Day 8. These findings confirm that the integration of antioxidant-rich components, particularly in formulation H, can substantially minimize lipid peroxidation and maintain the oxidative stability of chicken fillets during refrigerated storage.

The integration of these components resulted in films that significantly delayed spoilage processes. Formulation H, which included 15 % BE, 2 % chitosan, 2 % MSNPs, and 1 % CNC, consistently outperformed other formulations in all preservation metrics. This film demonstrated the lowest levels of lipid oxidation (measured by TBARS and FFA), protein oxidation (carbonyl content), and hydrogen peroxide accumulation. These effects can be attributed to the combination of phenolic radical scavenging, oxygen restriction by CNCs, and the sustained release mechanism of MSNPs (Criado et al., 2017; Litescu et al., 2014). Similarly, microbial spoilage indicators such as total volatile nitrogen (TVN) and total bacterial count (TBC) were lowest in formulation H. This was likely due to the synergistic action of chitosan's membrane-disrupting effect, the antimicrobial properties of phenolics, and the protection and delivery function of MSNPs (Doan et al., 2025).

### 3.9 Total Volatile Nitrogen (TVN)

TVN values, which reflect protein decomposition due to microbial and enzymatic activity, rose markedly in all treatments during the 8-day storage period (Fig. 4B). The Control group demonstrated the most dramatic increase, from 1.31 mg N/100 g on Day 1 to 26.13 mg N/100 g by Day 8 indicating advanced spoilage.

Formulations A and B exhibited similar trends, with TVN levels reaching 24.73 and 24.85 mg N/100 g, respectively, by Day 8. These films lacked chitosan and mesoporous silica, resulting in limited antimicrobial protection. By contrast, formulations containing chitosan showed significantly lower TVN accumulation. Formulation C (with 2 % chitosan and 10 % extract) reached 23.10 mg N/100 g, while formulation D reduced this to 21.23 mg N/100 g, showing moderate control of protein degradation. The best performance was observed in formulation H, which exhibited the lowest TVN rise reaching only 16.80 mg N/100 g by Day 8. This represents a 36 % reduction in TVN compared to the Control. The inclusion of chitosan, mesoporous silica, CNC, and 15 % plant extract in formulation H likely provided strong synergistic antimicrobial and antioxidant effects that delayed spoilage. These results confirm that TVN formation is effectively suppressed by active packaging films, especially those incorporating multiple bioactive components as in formulation H, thereby enhancing the shelf life of chicken fillets under refrigeration.

The suppression of TVN formation in active films, especially formulation H, can be explained by multiple interrelated mechanisms. Chitosan is known for its intrinsic antimicrobial activity, which disrupts microbial cell membranes and inhibits spoilage organisms (Ul-Islam et al., 2024). Additionally, the beet leaf extract contains phenolic compounds and flavonoids with proven antibacterial properties, which can interfere with microbial metabolism and enzyme activity (Platosz et al., 2020). Encapsulation of the extract in SBA-15 MSNPs protects these bioactives from environmental degradation and enables a sustained antimicrobial effect throughout storage. CNC enhances the structural density of the film, reducing oxygen and moisture permeability conditions favorable for microbial proliferation. (Babaei-Ghazvini et al., 2024) Collectively, these features contribute to a synergistic antimicrobial system that delays proteolysis and maintains protein integrity in refrigerated chicken fillets.

### 3.10 Hydrogen Peroxide Content

Hydrogen peroxide, a reactive oxygen species formed during oxidative spoilage, increased across all treatments over time (Fig. 4C). The Control group showed the highest accumulation, with levels rising from 0.342 μmol/g on Day 1 to 1.667 μmol/g by Day 8, indicating extensive oxidative deterioration. Formulations A and B, which lacked antioxidant-rich ingredients like chitosan and mesoporous silica, showed only slight improvements over the Control. By Day 8, their hydrogen peroxide levels reached 1.558 and 1.550 μmol/g, respectively. In contrast, formulations enriched with active components displayed better oxidative stability. Formulation C showed a Day 8 level of 1.500 μmol/g, while formulation D performed better, reducing hydrogen peroxide accumulation to 1.275 μmol/g. Formulation H achieved the greatest inhibition of hydrogen peroxide production, reaching only 1.175 μmol/g on Day 8 a 29 % reduction compared to the Control. Starting from 0.283 μmol/g on Day 1, formulation H consistently maintained the lowest levels throughout the storage period. These results suggest that antioxidant-rich films particularly those combining chitosan, mesoporous silica, CNC, and plant extract as in formulation H can substantially curb oxidative spoilage in chicken fillets, preserving their quality during refrigerated storage.

The enhanced oxidative stability of films containing chitosan, MSNPs, CNC, and encapsulated beet leaf extract can be attributed to multiple synergistic mechanisms. Chitosan acts as a natural antioxidant by chelating pro-oxidant metal ions and scavenging free radicals (Kim & Thomas, 2007). The phenolic compounds in the beet leaf extract, especially flavonoids and anthocyanins, contribute potent electron-donating capacity, neutralizing reactive oxygen species like H₂O₂ (Zahra et al., 2024). Encapsulation of these bioactives in SBA-15 MSNPs protects their functional integrity and ensures sustained release, while CNC improves the film's barrier properties, limiting oxygen ingress and moisture transfer both of which are critical factors in oxidative processes. Together, these components create a multifunctional packaging system that effectively mitigates oxidative spoilage in meat products.

### 3.11 Total Bacterial Count

Microbial growth, measured by total bacterial count (TBC), increased steadily in all samples throughout the 8-day refrigerated storage period (Fig. 4D). The Control group exhibited the highest bacterial load, starting from 2.93 log CFU/g on Day 1 and reaching 7.54 log CFU/g by Day 8, surpassing the general microbial spoilage threshold for raw poultry (typically 7 log CFU/g).

Formulations A and B, which lacked chitosan and mesoporous silica, showed similar bacterial growth to the Control, reaching 7.41 and 7.40 log CFU/g, respectively, by Day 8. This indicates that PVA films alone, even when combined with CNC and low extract levels, provided limited antimicrobial effects. On the other hand, films enriched with chitosan and extract (formulations C, D, and H) significantly inhibited bacterial proliferation. Formulation C reduced Day 8 TBC to 6.57 log CFU/g, and formulation D further lowered it to 6.36 log CFU/g.

Formulation H achieved the greatest microbial control, with TBC increasing from only 2.11 log CFU/g on Day 1 to 6.18 log CFU/g on Day 8 well below the spoilage limit and the Control group. This represents an approximate 18 % reduction in bacterial load compared to the Control at the end of storage. The antimicrobial effectiveness of formulation H is attributed to the synergistic action of chitosan (known for its intrinsic antimicrobial activity), mesoporous silica, CNC, and a high concentration of plant extract (15 %). These results clearly demonstrate that films incorporating multiple active agents particularly formulation H can significantly suppress microbial growth and effectively extend the shelf life of chicken fillets under refrigeration.

The strong antimicrobial effect observed in formulation H is attributable to the synergistic integration of multiple bioactive components. Chitosan, known for its polycationic nature, disrupts microbial cell membranes through electrostatic interactions, leading to leakage of cellular contents (Ul-Islam et al., 2024). The phenolic-rich beet leaf extract contributes additional antibacterial action by targeting microbial enzymes and interfering with membrane permeability (Bouarab-Chibane et al., 2019). Encapsulation within SBA-15 enhances the stability and sustained release of these bioactives, prolonging their inhibitory effects. Moreover, the inclusion of cellulose nanocrystals reinforces the film matrix, improving barrier properties that restrict moisture and oxygen diffusion key factors for microbial growth. Collectively, these mechanisms position formulation H as a highly effective bioactive packaging system for extending the microbial shelf life of poultry products under cold storage.

### 3.12 Color Indexes

**Fig. 5:**
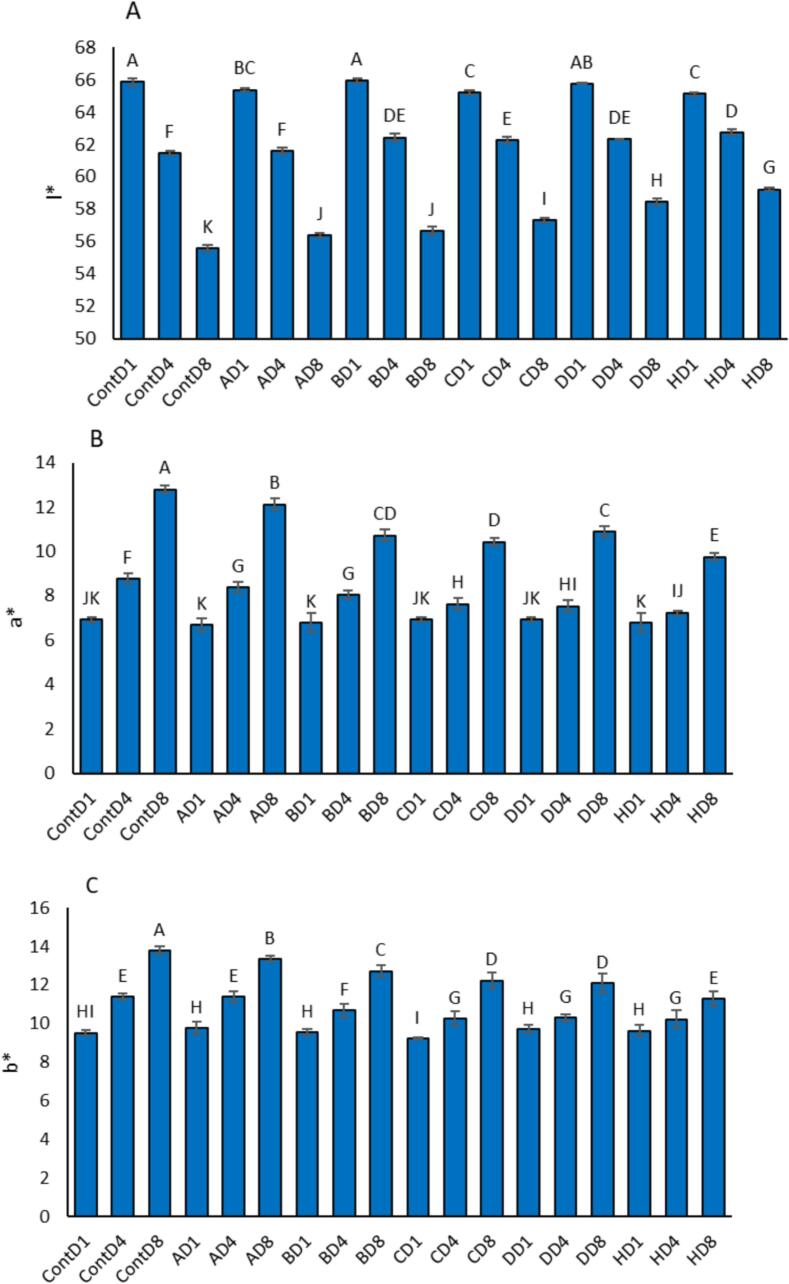
Effect of film composition on the color indexes (L*, a*, and b*) of chicken fillet during refrigerated storage (Days 1, 4, and 8) to evaluate their potential for shelf-life extension. The formulations included: Control (0 % of all components), A (17 % PVA, 0 % chitosan, 0 % mesoporous silica, 0.5 % CNC, 5 % extract), B (17 % PVA, 0 % chitosan, 0 % mesoporous silica, 1 % CNC, 10 % extract), C (17 % PVA, 2 % chitosan, 0 % mesoporous silica, 1 % CNC, 10 % extract), D (17 % PVA, 2 % chitosan, 2 % mesoporous silica, 0.5 % CNC, 15 % extract), and H (17 % PVA, 2 % chitosan, 2 % mesoporous silica, 1 % CNC, 15 % extract). Data are presented as mean ± standard error (SE) of three independent replicates (n = 3). Statistical differences among treatments were analyzed using one-way ANOVA followed by Duncan's multiple range test at p < 0.05. Different lowercase letters above the bars (or data points) indicate significant differences, confirming the reliability and reproducibility of the results.

Color is a key quality attribute of meat products, and changes in L* (lightness), a* (redness), and b* (yellowness) values were monitored during storage to evaluate oxidative and microbial degradation (Fig. 5). The Control group showed the most significant color deterioration, with L* decreasing from 65.84 (Day 1) to 55.59 (Day 8), a* increasing from 6.92 to 12.80, and b* rising from 9.53 to 13.77. These changes reflect the combined effects of myoglobin oxidation and surface dehydration.

Active films helped preserve the visual quality of the chicken fillets. Among them, formulation H exhibited the least deviation in all color parameters. Its L* value dropped slightly from 65.12 to 59.22, while a* increased from 6.78 to 9.75, and b* from 9.63 to 11.30 all lower than the Control group. These data suggest that formulation H effectively reduced oxidative browning and discoloration during storage. Other active formulations (C and D), which included chitosan and extract, also preserved color better than A and B. For example, formulation D showed an L* decline from 65.75 to 58.47, and a* rose from 6.93 to 10.90, while b* increased from 9.73 to 12.11 again, all lower than in the Control.

Formulations A and B, with less or no chitosan, had color shifts similar to the Control. Their a* and b* values increased to above 12, and L* dropped to approximately 56.4–56.7, indicating weaker protective effects. Films containing higher levels of antioxidant-rich extract (10–15 %) and barrier components such as chitosan and mesoporous silica were more effective in maintaining surface color, with formulation H again outperforming all others in preserving the fresh appearance of chicken fillets.

Color stability, an important sensory parameter, was also best maintained in active films containing beet extract. The natural pigments (betalains and anthocyanins) not only contributed to the initial color but also acted as antioxidants to prevent pigment degradation, particularly of myoglobin in meat (Khoo et al., 2017). Moreover, the films' protective barrier properties limited oxidative and enzymatic browning, as reflected in the minimal shifts in L*, a*, and b* values for formulation H (Sun et al., 2012; Zhu et al., 2024). Myoglobin oxidation primarily responsible for browning and a* elevation is mitigated by phenolic compounds present in beet leaf extract, which scavenge reactive oxygen species and chelate pro-oxidant metal ions (Olszowy, 2019). Chitosan provides an oxygen-restrictive matrix, further limiting oxidative processes. Moreover, SBA-15 MSNPs enhance the stability and controlled release of encapsulated antioxidants, ensuring prolonged protection. CNCs reinforce film integrity, reducing moisture loss and thereby preserving L* values (Daeialiakbar et al., 2025). Together, these synergistic effects explain the superior performance of formulation H in maintaining the surface color and visual appeal of chicken fillets during storage.

The ability of active films to stabilize pH during refrigerated storage can be attributed to the combined antimicrobial and barrier functions of chitosan, MSNPs, CNC, and phenolic-rich beet leaf extract. Chitosan's cationic nature enables electrostatic interaction with negatively charged microbial cell membranes, increasing membrane permeability and leading to leakage of intracellular constituents, thereby suppressing microbial metabolism responsible for amine and ammonia formation (Ul-Islam et al., 2024). Simultaneously, phenolic and flavonoid compounds in beet leaf extract, including betacyanins and coumarins, inhibit microbial proteases and decarboxylases that contribute to alkaline metabolite accumulation (Thiruvengadam et al., 2024). The encapsulation of these bioactives within mesoporous silica nanoparticles ensures sustained release and stability, maintaining antimicrobial activity throughout storage (Deng et al., 2024a). Furthermore, CNC reinforcement enhances film compactness, reducing gas permeability and moisture migration conditions favorable to microbial proliferation and enzymatic spoilage (Babaei-Ghazvini et al., 2024). This synergistic network effectively slows pH rise, as evidenced by the moderate increase observed in formulation H, which maintained the lowest pH fluctuation among all treatments.

A similar multi-mechanistic interplay explains the inhibition of lipid hydrolysis, as reflected by reduced FFA levels. The antioxidant-rich beet leaf extract acts as a primary free-radical scavenger, neutralizing lipid-derived radicals and stabilizing the cell membrane environment (Zahra et al., 2024). Encapsulation in MSNPs prevents rapid oxidation of these phenolics, ensuring their gradual diffusion and prolonged antioxidative protection during storage (Deng et al., 2024b). Chitosan's matrix further contributes by chelating transition metal ions (Fe^2+^, Cu^2+^) that catalyze lipid peroxidation, while CNC reinforcement restricts oxygen diffusion, thereby retarding both hydrolytic and oxidative degradation pathways (Kim & Thomas, 2007; Wang et al., 2020). The combination of these mechanisms in formulation H provided an optimal balance between antioxidant activity, structural integrity, and controlled bioactive release, resulting in lower FFA accumulation and better lipid stability compared with other formulations. Collectively, these results demonstrate that the multifunctional synergy among chitosan, CNC, MSNPs, and beet leaf extract not only limits microbial and enzymatic spoilage but also protects lipids from oxidative degradation, ensuring extended freshness of chicken fillets during refrigeration.

### 3.13 FT-IR Spectroscopy film

**Fig. 6:**
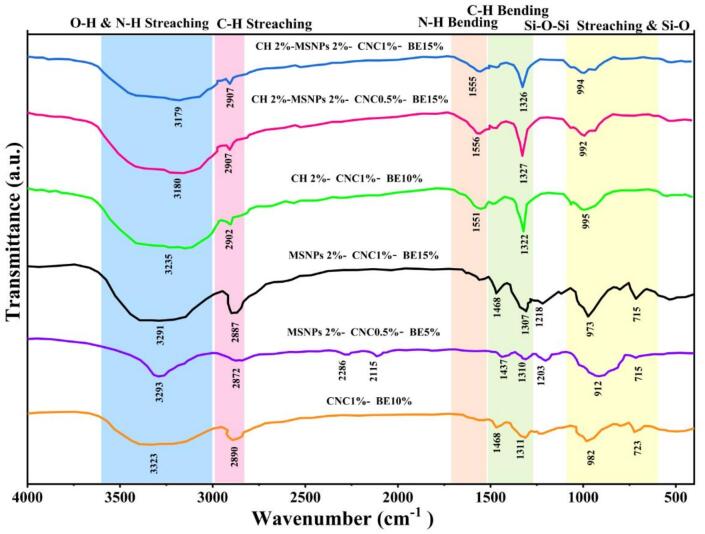
FT-IR spectra of composite films illustrating functional group interactions across different formulations with varying proportions of chitosan (CH), mesoporous silica nanoparticles (MSNPs), cellulose nanocrystals (CNC), and beet leaf extract (BE). The spectra highlight the influence of these components on the chemical interactions within the composite films.

The FT-IR spectra of the composite films (Fig. 6) revealed distinct absorption bands corresponding to the functional groups of PVA, CH, MSNPs, CNC, and BE. These spectra demonstrated notable variations in peak intensity and position, indicating chemical interactions and potential compatibility among the components. A broad absorption band observed in the range of 3200–3500 cm^−1^ in all formulations corresponds to –OH and –NH stretching vibrations, signifying the presence of hydroxyl and amine groups from PVA, chitosan, CNC, and plant extract. The intensity of this band was more pronounced in formulations containing chitosan and CNC, suggesting enhanced hydrogen bonding among components.

The characteristic peaks around 1630–1650 cm^−1^ were attributed to amide I (C=O stretching) from chitosan and proteinaceous compounds in the extract. Notably, formulations with higher chitosan and extract content (especially formulations D and H) displayed more prominent peaks in this region, indicating stronger intermolecular interactions. Absorption bands near 1000–1100 cm^−1^ were assigned to Si–O–Si stretching from the mesoporous silica nanoparticles. These peaks were clearly visible in formulations D and H, confirming the successful incorporation of MSNPs into the matrix. The spectra also exhibited shifts in fingerprint regions (600–1500 cm^−1^), which may reflect complex interactions between CNC, MSNPs, and BE with the PVA matrix. These shifts were most notable in formulation H, suggesting strong compatibility and crosslinking among all bioactive components. FT-IR analysis confirmed the successful integration of functional components into the film matrix and indicated enhanced molecular interactions, particularly in multi-component systems like formulation H. These interactions likely contribute to the improved mechanical and barrier properties of the films, which support their effectiveness in food preservation applications.

### 3.14 Scanning Electron Microscopy (SEM) of film

**Fig. 7:**
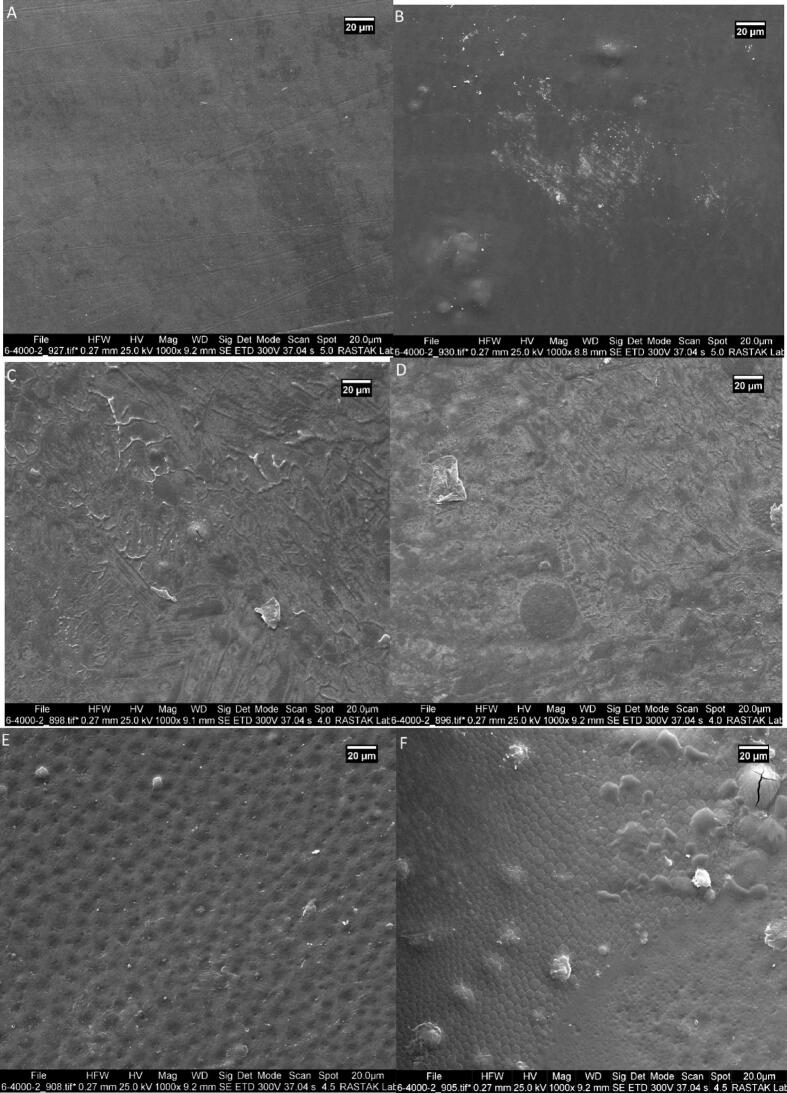
Scanning Electron Microscopy (SEM) images of composite films illustrating surface morphology and structural characteristics for different formulations with varying proportions of chitosan (CH), mesoporous silica nanoparticles (MSNPs), cellulose nanocrystals (CNC), and beet leaf extract (BE). The sample compositions are as follows: (A) 0 % CH, 0 % MSNPs, 1 % CNC, 10 % BE; (B) 0 % CH, 2 % MSNPs, 0.5 % CNC, 5 % BE; (C) 0 % CH, 2 % MSNPs, 1 % CNC, 15 % BE; (D) 2 % CH, 0 % MSNPs, 1 % CNC, 10 % BE; (E) 2 % CH, 2 % MSNPs, 0.5 % CNC, 15 % BE; and (F) 2 % CH, 2 % MSNPs, 1 % CNC, 15 % BE. These images provide insights into the impact of component variations on film structure, porosity, and overall morphology.

SEM analysis (Fig. 7) was conducted to investigate the surface morphology and structural characteristics of the composite films with varying concentrations of chitosan (CH), mesoporous silica nanoparticles (MSNPs), cellulose nanocrystals (CNC), and beet leaf extract (BE). The micrographs reveal notable differences in texture, homogeneity, and porosity depending on the formulation. Formulations A, B, and C, which lacked chitosan, generally exhibited rougher and more irregular surfaces. In particular, sample A (0 % CH, 0 % MSNPs, 1 % CNC, 10 % BE) displayed a heterogeneous and somewhat porous surface, indicative of poor matrix cohesion. Sample B (0 % CH, 2 % MSNPs, 0.5 % CNC, 5 % BE) exhibited more granular structures, likely due to the presence of MSNPs without the stabilizing effect of chitosan. Sample C (0 % CH, 2 % MSNPs, 1 % CNC, 15 % BE) revealed more densely packed domains but still lacked surface smoothness.

In contrast, the addition of chitosan in formulations D, E, and F resulted in visibly smoother and more compact surfaces. Sample D (2 % CH, 0 % MSNPs, 1 % CNC, 10 % BE) showed a relatively uniform surface with fewer voids, suggesting improved film integrity due to hydrogen bonding between chitosan and CNC. Sample E (2 % CH, 2 % MSNPs, 0.5 % CNC, 15 % BE) exhibited a moderately smooth and continuous structure, while sample F (2 % CH, 2 % MSNPs, 1 % CNC, 15 % BE) demonstrated the most homogeneous and dense morphology among all, with minimal surface defects. These observations indicate that chitosan plays a critical role in enhancing film uniformity and reducing porosity, especially when combined with MSNPs and CNC. The structural compactness observed in formulation F correlates with its superior performance in physicochemical and microbiological stability tests, supporting its potential as an effective active packaging material.

Compared with other active packaging systems reported in the literature—such as chitosan films containing free essential oils, phenolic extracts, or metallic nanoparticles—the bioactive chitosan-based film developed in this study exhibited superior multifunctional performance. Conventional chitosan–plant extract films often suffer from poor compatibility between hydrophilic polymer matrices and hydrophobic bioactives, leading to rapid release and short-lived antioxidant effects (Kumar et al., 2020). In contrast, the integration of beet leaf extract encapsulated within SBA-15-type mesoporous silica nanoparticles enabled controlled and sustained release of phenolics, maintaining antioxidant and antimicrobial activity throughout storage. This synergistic matrix effectively combined chemical antioxidant mechanisms (radical scavenging and metal chelation) with physical barrier effects (via chitosan–CNC interactions), controlled release of active compounds (from MSNPs), and direct antimicrobial action from both chitosan and phenolic constituents. The multifactorial interplay of these mechanisms resulted in markedly enhanced oxidative and microbial protection, reflected in lower TBARS, TVN, protein oxidation, and microbial counts compared with similar chitosan-based or essential-oil-loaded films reported for meat preservation. Collectively, this system demonstrated a breakthrough by uniting encapsulation technology, biopolymer reinforcement, and plant-based antioxidant delivery, achieving a longer shelf life and better physicochemical stability of chicken fillets than previously established active packaging approaches.

## 4 Conclusion

This study successfully developed multifunctional, biodegradable edible films incorporating beet leaf extract (BE) encapsulated in mesoporous silica nanoparticles (MSNPs) and reinforced with cellulose nanocrystals (CNCs) and chitosan (CH). Chemical profiling of the beet leaf extract revealed diverse bioactives phenolic acids, flavonoids, coumarins, and anthocyanins exhibiting strong antioxidant and antimicrobial potential. The physicochemical characterization of MSNPs confirmed their suitability as an efficient nanocarrier for stabilizing and sustaining the release of BE. Film formulations with varying ratios of CH, CNC, MSNPs, and BE were systematically compared to identify the optimal composition based on mechanical strength, barrier efficiency, and bioactive release performance. Among these, formulation H (17 % PVA, 2 % CH, 2 % MSNPs, 1 % CNC, 15 % BE) demonstrated the most balanced and superior characteristics, including low water vapor and oxygen transmission rates, uniform microstructure, and the highest antioxidant and antimicrobial efficacy. When applied to chicken fillets, formulation H most effectively reduced lipid and protein oxidation, stabilized pH, and suppressed microbial proliferation during refrigerated storage, while preserving the meat's color and freshness. The enhanced performance of formulation H reflects the synergistic interplay between chitosan's antimicrobial barrier, CNC's structural reinforcement, MSNP-mediated controlled release, and the potent antioxidant capacity of BE. Overall, this optimized film represents a promising active packaging platform that not only extends the shelf life and safety of perishable foods but also supports agricultural waste valorization, environmental sustainability, and clean-label packaging innovation. Future research should focus on scaling up production and tuning the release kinetics for broader food preservation applications.

## CRediT authorship contribution statement

**Solmaz Pourbarghi Soufiani:** Writing – original draft, Validation, Software, Methodology. **Shima Yousefi:** Writing – review & editing, Writing – original draft, Supervision, Project administration, Investigation, Funding acquisition. **Masoud Honarvar:** Writing – review & editing. **Weria Weisany:** Writing – review & editing, Writing – original draft, Validation, Software, Methodology. **Gholamhassan Asadi:** Writing – review & editing.

## Declaration of competing interest

The authors declare that they have no known competing financial interests or personal relationships that could have appeared to influence the work reported in this paper.

## Data Availability

Data will be made available on request.
